# The startle reflex in echolocating odontocetes: basic physiology and practical implications

**DOI:** 10.1242/jeb.208470

**Published:** 2020-03-12

**Authors:** Thomas Götz, Aude F. Pacini, Paul E. Nachtigall, Vincent M. Janik

**Affiliations:** 1 Sea Mammal Research Unit, Scottish Oceans Institute, School of Biology, University of St Andrews, Fife KY16 8LB, UK; 2 Hawaii Institute of Marine Biology, University of Hawaii at Manoa, P.O. Box 1346, Kaneohe, Hawaii 96744, USA

**Keywords:** Acoustic startle reflex, Hearing thresholds, Startle thresholds, Rise time, Anthropogenic noise, Bottlenose dolphin, *Tursiops*, *Pseudorca*, Brainstem

## Abstract

The acoustic startle reflex is an oligo-synaptic reflex arc elicited by rapid-onset sounds. Odontocetes evolved a range of specific auditory adaptations to aquatic hearing and echolocation, e.g. the ability to downregulate their auditory sensitivity when emitting clicks. However, it remains unclear whether these adaptations also led to changes of the startle reflex. We investigated reactions to startling sounds in two bottlenose dolphins (*Tursiops truncatus*) and one false killer whale (*Pseudorca crassidens*). Animals were exposed to 50 ms, 1/3 octave band noise pulses of varying levels at frequencies of 1, 10, 25 and 32 kHz while positioned in a hoop station. Startle responses were quantified by measuring rapid muscle contractions using a three-dimensional accelerometer attached to the dolphin. Startle magnitude increased exponentially with increasing received levels. Startle thresholds were frequency dependent and ranged from 131 dB at 32 kHz to 153 dB at 1 kHz (re. 1 µPa). Startle thresholds only exceeded masked auditory AEP thresholds of the animals by 47 dB but were ∼82 dB above published behavioural audiograms for these species. We also tested the effect of stimulus rise time on startle magnitude using a broadband noise pulse. Startle responses decreased with increasing rise times from 2 to 100 ms. Models suggested that rise times of 141–220 ms were necessary to completely mitigate startle responses. Our data showed that the startle reflex is conserved in odontocetes and follows similar principles as in terrestrial mammals. These principles should be considered when assessing and mitigating the effects of anthropogenic noise on marine mammals.

## Abstract (precis)

**Summary:** The acoustic startle reflex is conserved in echolocating toothed whales and should be considered when predicting marine mammal responses to human-generated underwater noise.

## INTRODUCTION

The acoustic startle response is a rapid contraction of flexor muscles (flinch) which is mediated by an oligo-synaptic reflex arc in the brainstem (Koch and Schnitzler, 1997). The reflex arc involves the cochlear nucleus and the caudal pontine reticular formation (PnC) in the brainstem before activating motoneurons in the spine. Acoustic activation of the reflex generally requires sound stimuli to have two parameters: (1) a minimum sensation level, i.e. a stimulus must exceed the auditory threshold by 70–90 dB (Pilz et al., 1987; Stoddart et al., 2008), and (2) a rapid onset time (rise time), traditionally considered to be less than 15–25 ms (Blumenthal and Berg, 1986; Fleshler, 1965).

**Symbols and abbreviations**

AEP: auditory evoked potential
β: model coefficient for a given predictor variable or factor, in a model with a link function from the exponential family e^β^ represents the coefficient on the scale of the response variable (see also Table S1)
e: Euler's number (∼2.718)
FA: focal animal
p–p VeDBA: maximum peak to peak vectorial dynamic body acceleration
Jerk: maximum of the norm of the differentiated acceleration record d**A**/d*t* scaled to give results in m s^−3^. **A** is a 3-column vector obtained from a triaxial accelerometer.
SPL: sound pressure level (dB re. 1 µPa; studies on terrestrial mammals are quoted in dB re. 20 µPa; sensation levels in dB re. hearing threshold.)
OA: ‘other animal’ (non-focal)
RL: received level, i.e. the sound pressure level (in dB re. 1 µPa) at the dolphin's ear
SEL: sound exposure level, the integral of the squared sound pressure over time (in dB re. 1 µPa^2^)

The startle reflex has been a model system in behavioural physiology and neuroscience for several decades, with both rodent (full body or neck flinch) and human models (eyeblink) being studied intensively. The reflex arc is used as a model for simple learning mechanisms, the functioning of sensory motor gating and emotional processing (Koch and Schnitzler, 1997; Lang et al., 1998). The latter is the result of startle magnitude being modulated by emotional state, i.e. watching pleasant/unpleasant images (humans) or inducing conditioned fear in animal models (Lang et al., 1998). These processes are mediated through efferent projections from higher order brain centres, namely the amygdala (Koch and Schnitzler, 1997). While most research has focused on the behavioural correlates related to these efferent pathways, repeated startle elicitation also seems to modulate emotional state through an afferent pathway (Götz and Janik, 2011). In a study that investigated follow-up responses after repeated startle elicitation, the majority of tested grey seals (*Halichoerus grypus*) sensitised to the stimulus, exhibited flight responses, developed place avoidance and showed signs of temporary fear conditioning (Götz and Janik, 2011). These findings kindled research interest in the startle reflex in the context of practical applications such as target-specific predator deterrence from farmed fish stocks and fisheries (Götz and Janik, 2015, 2016; Schakner et al., 2017). Furthermore, this reflex arc is relevant for understanding the physiological mechanisms underlying aversive responses to anthropogenic ocean noise in marine mammals (Harris et al., 2018), but empirical data is lacking.

Brief, ‘startle-like’ movement responses have been previously described in harbour porpoises *Phocoena phocoena* (Kastelein et al., 2012). However, the functioning of the reflex arc has not been investigated in any odontocete species in an experimental setup that allows researchers to distinguish between generic movement responses and startle elicitation. Unequivocal evidence for a startle response requires objective quantification of startle magnitudes within measurable latency windows (e.g. Pilz et al., 1987). Odontocetes possess sensitive underwater hearing with sophisticated sensory gating abilities that enable them to solve complex echolocation tasks (Nachtigall et al., 2000). Most delphinids regularly produce broadband, high-intensity echolocation clicks at source levels that can exceed 225 dB re. 1 µPa [peak to peak (p–p)] (Madsen and Surlykke, 2013). Some *odontocetes* also have the ability to reduce forward masking by both reducing the intensity of the outgoing clicks (automatic gain control) and using a presumed neuronal gain control mechanism (Nachtigall and Supin, 2008; Supin et al., 2005). In animals performing an echolocation task, auditory evoked potentials (AEPs) in the brainstem remained constant in spite of echo levels decreasing either as the result of increasing target distance or decreasing target strength (Nachtigall and Supin, 2008; Supin et al., 2005). Perhaps even more strikingly, odontocetes also seem to downregulate their auditory sensitivity after a warning sound (Nachtigall and Supin, 2015; Nachtigall et al., 2016a, 2018). This mechanism is most likely controlled by conditioning processes since expectancy reduces the effect, whereas an unpredictable latency between the warning and test sound increases it (Nachtigall et al., 2016b). Taking all of these mechanisms into account, it remains unclear whether the startle reflex is present in odontocetes and whether the physiological pathway has been modified in some way as an adaptation to the evolution of an active, high-intensity biosonar system. In this study, we therefore investigated whether the startle reflex is present in odontocetes and set out to quantify the effects of the two most important sound stimulus parameters that determine the reflex in terrestrial mammals: stimulus amplitude (sound pressure level) and rise time.

## MATERIALS AND METHODS

### Experimental procedures

Experiments were carried out with trained captive animals at the research facility of the Hawaii Institute of Marine Biology on Coconut Island in Kane'ohe Bay in 2012. The test subjects were a 26 year old female (BJ) and a ∼21 year old male (Boris) bottlenose dolphin [*Tursiops truncatus* (Montagu 1821)] and a >35 year old false killer whale [*Pseudorca crassidens* (Owen 1846)] (Kina, exact age unknown) which were held in sea pens at the institute. The work was completed under a Marine Mammal Permit issued to P.E.N. from the National Marine Fisheries Service (NMFS) office of Protected Species with protocols approved by the Institutional Animal Care and Utilization Committee of the University of Hawaii at Manoa.

**Fig. 1.**
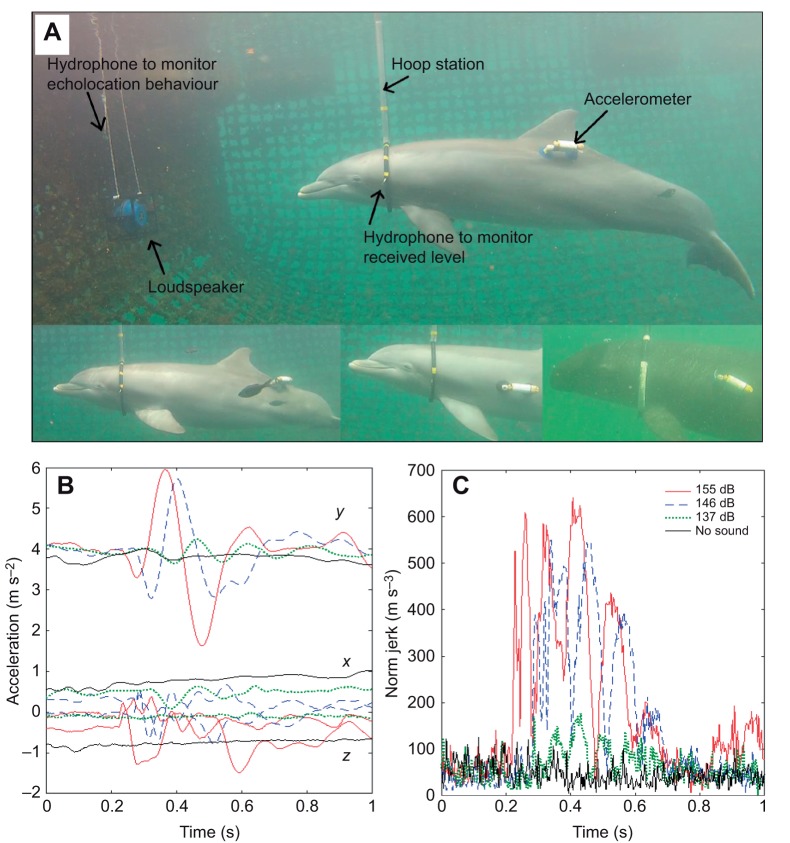
**Experimental setup and quantification of startle responses.** (A) Experimental setup and example tag attachment locations. (B) Accelerometer recording of a startle response at different received levels (RLs; coloured lines) and baseline movement during the no-sound control in black. The three labelled traces for each condition represent the three spatial axes (*x*,*y*,*z*) of the triaxial accelerometer. The *y*-axis offset is the result of gravity-induced ‘static acceleration’. The start of the sound pulse coincides with 0 s on the timescale. (C) Startle responses quantified by the norm jerk (coloured lines), i.e. the norm of the differential (time derivate) of the acceleration record at different RLs (colours) and during the no-sound control (black).

Hearing thresholds for all animals were determined routinely and had been measured prior to the experiments using the AEP method as described in Pacini et al. (2011). These thresholds were masked thresholds because of the high snapping shrimp noise in Kane'ohe Bay. The animals were trained to enter a hoop station which enabled them to remain stationary in front of the sound projector (Fig. 1A). A data logger which consisted of a three-dimensional accelerometer sensor (GCDC X 6-2, Gulf Coast Data Concepts LLC, Waveland, MA, USA) was placed in a custom-made underwater housing, attached with suction cups laterodorsally to the animal and used to quantify brief muscle flinches typically associated with the startle reflex (Fig. 1A). The accelerometer sampled 320 data points s^−1^ with a maximum dynamic range of ±6 g (±58.8 m s^−2^) and a resolution of 16 bit (0.00006 g/counts). In addition, the behaviour of the animal was monitored with a GoPro HD Hero 2 underwater camera positioned laterally of the animal. Signals at 1 and 10 kHz were projected with a Lubell loudspeaker 9162T (Lubell Labs Inc., Columbus, OH, USA) that was positioned 1.5 m in front of the hoop station (Fig. 1A). An ITC 1032 transducer (Gavial ITC, Santa Barbara, CA, USA) was used in place of the Lubell speaker for projection of signals at ultrasonic frequencies of 25 and 32 kHz. The digital signals were played through a National Instruments (NI) DAQ card (USB 6251 M) controlled by LabView software (National Instruments, Austin, TX, USA) on a laptop computer. Output from the NI card was fed through an Etec ATN127 signal attenuator (Etec, Frederiksvaerk, Denmark) onto a Hafler P3000 ‘trans nova’ power amplifier (Hafler, Port Coquitlam, BC, Canada), which was connected to the respective sound transducer. In addition to the playback setup, two monitoring hydrophones were installed (a B&K 8103, BRÜEL & KJÆR, Nærum, Denmark and a Reson TC 4103, RESON, Slangerup, Denmark). The Reson hydrophone was placed on the hoop station and the B&K hydrophone was placed ∼30 cm above the sound projector to monitor echolocation behaviour. The hydrophone located at the hoop station was used to monitor received levels (RLs) in proximity to the dolphin's ear. The output of the two hydrophones was amplified with two Etec pre-amplifiers (Etec A1001), digitized with an additional National Instruments DAQ card (NI USB 6351 S, National Instruments) at sampling rates of 300 or 400 kHz and recorded as wav files onto another laptop computer.

The accelerometer was synchronized with the acoustic recording by applying three distinctive taps with the hydrophone against the stationary accelerometer. An experimental trial started with the dolphin positioning on a touch pad in front of the trainer. The trainer gave the dolphin a signal to enter the hoop station while the behaviour of the animal was monitored with an underwater camera connected to an LCD screen. Once the animal had settled into the hoop station, a countdown to the start of the playback began. The time between start of the countdown and the playback was varied randomly across trials with intervals ranging from 2 to 58 s. The animal was then called back with an acoustic signal (trainer whistle), returned to the touch pad and received a food reward. The reward remained the same throughout all trials irrespective of treatment and RL to avoid bias.

**Fig. 2.**
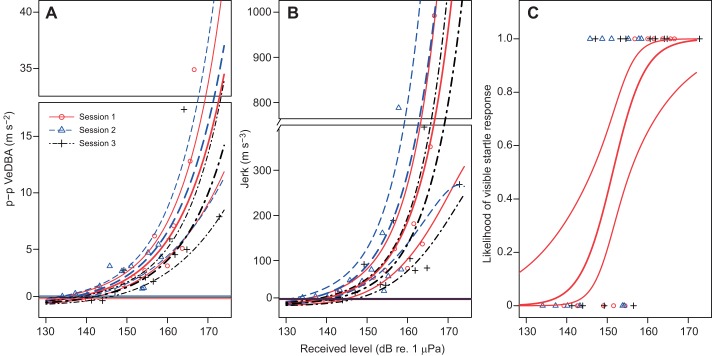
**Comparison of the three response metrics that were tested to quantify startle magnitude and determine startle thresholds.** (A) p–p VedBA obtained from the accelerometer record. (B) Maximum norm jerk (jerk), i.e. the norm of the time derivative of acceleration obtained from the differentiated acceleration record. (C) Presence or absence of a visually detectable startle flinch (yes/no) from video data. The curves represent predicted values and 95% confidence intervals for startle magnitude obtained from the gamma GLMs (in A,B) and the response probability (video data) obtained from the logistic GLM (in C). The horizontal lines represent average baseline acceleration during a no-sound control for each playback session. The startle threshold is defined as the RL where the predicted values cross these lines (A,B) or as a 50% response probability (C). Gap plots were used to allow visibility of all data points. Data from BJ (10 kHz test frequency).

**Fig. 3.**
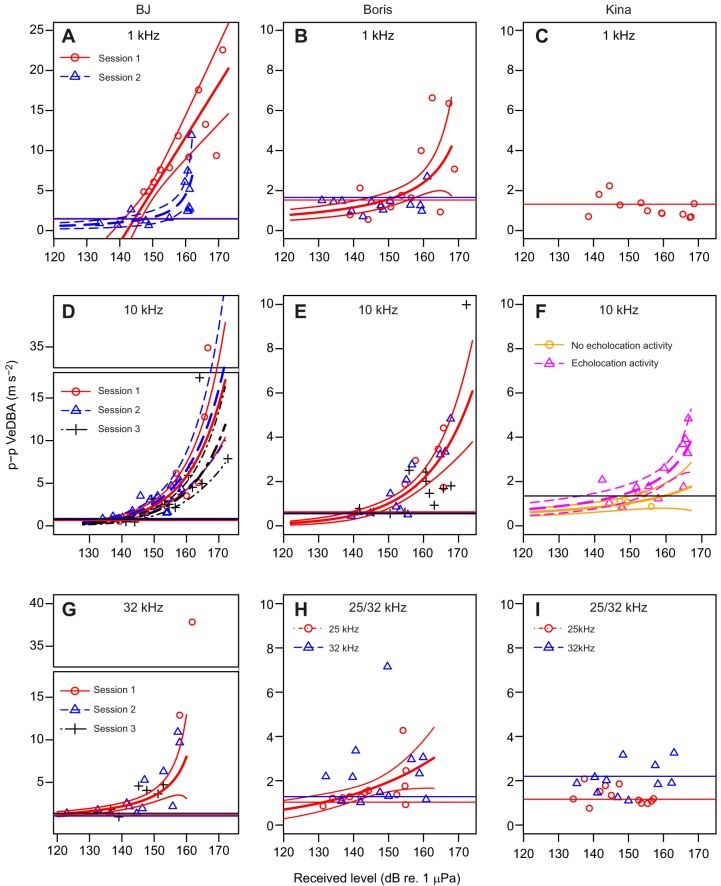
**Maximum peak to peak acceleration (VeDBA) in the 1 s time window after the onset of the sound as a function of received levels (in dB re. 1 µPa) for all playback sessions.** Maximum p–p VeDBA for all playback sessions at 1 kHz (A–C), 10 kHz (D–F), 32 kHz (G) and 25/32 kHz (H,I) for two bottlenose dolphins [*Tursiops truncatus*; BJ (A,D,G) and Boris (B,E,H)] and a false killer whale [*Pseudorca crassidens*; Kina (C,F,I)]. The curves represent predicted values and 95% confidence intervals obtained from the GLMs. If only one line (red, solid) of predicted values is plotted this indicates that playback session was not part of the model (except for Boris at 25 kHz where only one session was conducted). If RL was not significant, no trend line was plotted. The horizontal lines represent the average p–p VeDBA during the no-sound controls (trial with no playback) for each session. Plotting symbols depict playback session number (circles: 1, triangles: 2, crosses 3) except for F where symbols depict whether a non-focal animal (OA) emitted echolocation clicks during the respective trial (circle: no clicks emitted, triangle: clicks emitted). In some panels, gap plots were used to show all data points.

Two separate experiments were conducted. Experiment 1 was aimed at determining startle thresholds by quantifying the relationship between RL and startle magnitude. Experiment 2 was aimed at measuring the effect of rise time on startle magnitude while RLs were kept constant. Each experimental session typically consisted of 12 sound exposure trials and one random no-sound control during which exactly the same experimental procedure was followed but no playback was carried out. The tested sound stimuli in experiment 1 were 1/3 octave band noise pulses of 50 ms duration with a frequency centroid of 1, 10, 25 and 32 kHz and an onset time of 1–2 ms. The accelerometer tag was either positioned latero-cranial (10 kHz, Fig. 1A, centre and right inset panel) or dorso-caudal (1 kHz, 25 kHz and 32 kHz, Fig. 1A, top, and left inset panel). In a typical session of the threshold experiment (experiment 1), the sound pressure level was digitally decreased in 6 dB steps from the first trial towards the 6th trial, then increased by 3 dB and consecutively increased again in 6 dB steps up to the 12th trial using the Etec ATN127 Universal Signal Attenuator [see Figs 2 and 3 for actual sound pressure levels (SPLs)]. Some playback sessions contained fewer trials owing to malfunction of equipment or a decision by the trainer to abort the session. Overall, 20 sessions were conducted in experiment 1 across all test specimens and frequencies. SPL increments at the animal varied slightly as a result of sound propagation. Three playback sessions were conducted with BJ at 10 kHz (36 trials), two sessions at 1 kHz (25 trials) and three sessions at 32 kHz (20 trials). The first session for BJ at 32 kHz only consisted of two trials. Boris completed two sessions at 10 kHz (29 trials), two at 1 kHz (25 trials), one at 32 kHz (12 trials) and one at 25 kHz (12 trials). Two sessions were carried out with Kina at 10 kHz (18), one at 1 kHz (12 trials), one at 25 kHz (12 trials) and one at 32 kHz (11 trials).

In experiment 2, broadband noise pulses with rise and fall times of 2, 20 and 100 ms were tested. Digital pulses had the same average RMS SPL during the flat central section (plateau). Pulses were also normalised to have roughly the same sound exposure level (SEL), a metric that is related to the acoustic energy of the signal and constitutes the area under the curve of the signal's envelope. The digital envelope functions were set to result in signal rise and fall times of 2, 20 and 100 ms (with mild tapering) and durations of 140 ms, 158 ms and 238 ms with the plateau sections having respective durations of 136 ms, 118 ms and 38 ms. However, since band-limited noise is a stochastic signal with random amplitude fluctuations, these numbers should be considered as approximate values with regard to the actual playback signal. The −20 dB points of the broadband pulse in units of power spectral density were at ∼6.8 kHz and ∼19 kHz. Mean received levels (RMS) measured at the monitoring hydrophone at the hoop across all trials in experiment 2 were 165 dB re. 1 µPa for BJ, 162 dB re. 1 µPa for Boris and 158 dB re. 1 µPa for Kina. In experiment 2, two playback sessions were conducted with Boris (24 trials), two with BJ (24 trials but two trials could not be included in the analysis owing to a clipping problem on the accelerometer) and one with Kina (12 trials).

### Data analysis

Data analysis involved calculating RLs and time of playback from the calibrated acoustic record. Startle response magnitude was quantified by measuring various metrics using custom-written routines in MATLAB R 2011 (MathWorks, Inc., Natick, MA, USA). A 1 s time window was set after the onset of the sound pulse within which the various metrics were calculated. The same procedure was carried out for the ‘no-sound control’ trial, where five 1 s time windows were pseudorandomly selected. If no acoustic record was available for a session (*n*=2) because of equipment malfunction, the startle flinches were selected manually and the window set accordingly. The three-dimensional acceleration record (*A*) was high-pass filtered at 2 Hz (FIR filter) to remove slow movement responses prior to any further analysis. The p–p acceleration was then measured individually on all three channels (*x*,*y*,*z*) and the maximum vectorial dynamic body acceleration (VeDBA) was calculated as 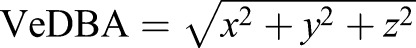 with *x*, *y* and *z* being the p–p values on each of the three accelerometer axes within the 1 s time window. This particular implementation of p–p VeDBA performed well in an initial exploratory data analysis. In addition, we tested the jerk as a metric which is given by the time derivative of acceleration and represents the rate of change of acceleration. The norm jerk was calculated by differentiating **A** (d**A**/d*t*) for each of the three axes of the accelerometer and obtaining the total as the norm of the triaxial jerk, i.e. the square-root of the sum of the squared values at a given time (Ydesen et al., 2014). This is referred to as the ‘total jerk’ or ‘norm jerk’ in Ydesen et al. (2014) and constitutes a one-dimensional vector (see Fig. 1C). The max norm jerk was then determined as the peak value within the 1 s long analysis window and is referred to in this paper as ‘max(imum) norm jerk’ or short ‘jerk’. For one animal (BJ) at one frequency (10 kHz), a visual threshold estimation method was tested in which one of us (V.M.J.) scored the visible occurrence of a ‘startle flinch’ in muted GoPro videos (see also Movie 1 for an example video of a startle flinch). We present a detailed comparison of these three startle response metrics for the test condition with the most comprehenesive data set (BJ at 10 kHz) in the results (Fig. 2). These are p–p VeDBA, maximum norm jerk and the binary scoring of videos (startle yes/no). These results also provide the rationale for why p–p VeDBA was taken forward for the main analysis (thresholds, rise time). We also show the results from Fig. 2A again in Fig. 3D to allow a direct comparison to other specimens and test frequencies (using the p–p VeDBA metric).

SPLs measured by the monitoring hydrophone at the hoop station were calculated as SPL=|*T*_x_|−Gain+20log(*V*) with |*T*_x_| denoting the absolute of the voltage sensitivity of the hydrophone (in dB re. 1 V Pa^−1^), Gain represents any gain in the recording chain (preamplifier, NI card) in dB and *V* is the voltage or amplitude measured in arbitrary units on a linear scale (Au, 1993). The voltage sensitivity values for the hydrophone were obtained by measuring values on the calibration chart at the respective test frequencies (−211.63 dB re. 1 V Pa^−1^ at 1 kHz, −211.55 dB re. 1 V Pa^−1^ at 10 kHz, −211 dB re. 1 V Pa^−1^ at 25 kHz, −211.3 dB re. 1 V Pa^−1^ at 32 kHz). If no acoustic record was available for a session (*n*=2) or part of a session (*n*=3), calibration data were used to estimate the RLs the animal was exposed to. The received levels (SPLs) measured at the hoop station were compensated for the transmission loss between the acoustic window of the dolphin (Popov and Supin, 1990; Popov et al., 2008) and the location of the monitoring hydrophone. This was achieved by using underwater video images from the respective playback sessions and assuming spherical spreading over the relatively short distance. Compensation values used for 1 and 10 kHz trials were +0.6 dB (BJ), +0.7 dB (Boris) and +1.95 dB (Kina). Compensations used for 23 and 32 kHz trials were +1.3 dB (BJ), +1.4 dB (Boris) and +1.95 dB (Kina).

An analysis of echolocation clicks was conducted for the recordings 10 s prior to sound exposure. Clicks were ascribed to the test subject of the respective trial (‘focal animal’, FA) if all of the following criteria were met: (1) the peak amplitude of the clicks was at least 30 dB higher at the hydrophone in front of the focal animal compared with the hydrophone at the hoop station; (2) clicks recorded at the hoop station showed clear off-axis characteristics in the recordings from the hoop station (see Au et al., 2012, clicks at ∼90 angles); and (3) the time of arrival difference between the two hydrophones was consistent with clicks coming from the test subject in the hoop station. If clicks did not match these criteria they were ascribed to other animals (OA) in the facility.

### Statistical modelling

In the following analysis, we used a well-established extension of the linear model, the generalized linear model (GLM), which allowed the use of non-linear link functions and non-gaussian error distributions (e.g. Zuur et al., 2009). GLMs are more flexible and preferable to traditional methods (data transformations) as they allow the *x*–*y* relationship (link function) and variance relationship (variance function) to be modelled separately (e.g. Zuur et al., 2009). We chose to fit GLMs with a gamma distribution of errors and either a log-link or inverse (hyperbolic) link function in R 3.4.0 (https://www.r-project.org/) to analyse data from the threshold experiment. The gamma distribution was best suited as it allowed to accommodate a wide range of non-symmetrical (skewed) data distributions for continuous, positive data values. The models always included p–p VeDBA (in m s^−2^) and the maximum norm jerk (BJ 10 kHz only) as independent variables and RL as a dependent variable. A model selection process was conducted using the corrected Akaike Information Criterion (AICc) for small sample sizes (‘MuMIn’ package for R; https://cran.r-project.org/web/packages/MuMIn/index.html) and the model with lowest AICc was selected (Barton, 2016). Model selection was purely based on this information criterion, balancing model fit against simplicity, and variables that did not reach significance were nevertheless retained in the final model. However, we refrained from plotting predicted values and calculating thresholds if the effect of the variable that was the primary purpose of the experiment [RL or rise time] was not significant (*P*<0.05). We report measures of effect size (coefficients), predicted values and associated uncertainty (confidence intervals) for all variables and factors of the two main experiments (see Table S1) instead of solely relying on an arbitrary significance level convention for *P*-values. Two possible link functions, a logarithmic link (to the base of Euler's number) or an inverse (hyperbolic) link were tested. The following additional predictor variables and factors were considered as candidates: playback session number as a factor, trial number (reflecting playback number) or the natural logarithm of trial number (within sessions) as a covariate. Trial number and its logarithm control for ‘within session habituation’, i.e. a decrease in response magnitude with repeated exposure (each additional trial). Playback session number controls for between session habituation but also accelerometer tag location at attachment sites were consistent within one session but not always across sessions. Echolocation activity by the focal animal (FA) and animals in neighbouring pens (OA) during a 10 s period prior to sound exposure were also considered as binary factors. In order to avoid overfitting, the factor OA was only tested for frequencies and subjects for which sufficient data were available, i.e. if a full acoustic and accelerometry record was available with data originating from at least two playback sessions at a given frequency (Kina 10 kHz, BJ 32 kHz and Boris at 1 kHz). FA click production was only considered as a factor if the stationing animal produced clicks in more than 2 trials and the previously described conditions were met regarding availability of sufficient data (which was only the case for BJ at 32 kHz). If trial (playback) number was included as a covariate in the final model, then predicted values for startle magnitude (curves in Figs 2 and 3) were calculated for an intermediate trial number (trial 6.5). Model assumptions were checked by qualitatively assessing residuals and *QQ* (quantile–quantile) plots. We deviated from this general procedure in only one case (BJ at 1 kHz) in which the model fitted poorly. In this case, there was some evidence for violation of the model assumptions (patterning of residuals) and the relationship between startle magnitude (max. p–p VeDBA) and RL appeared linear in the first but not the second session. We therefore fitted separate models for the first session (linear ‘identity’ link) and the second session (inverse link).

Predicted values for the modelled relationship between RL and startle magnitude were calculated using the predict function and a data frame with 0.1 decimal steps in R. These predicted values were plotted on the scale of the response variable in respective graphs. The startle threshold was defined as the RL (in the predict frame), which corresponded to the first predicted (fitted) value above the average ‘no sound control’ across all sessions (if session was not retained in the final model) or the no-sound control of the respective playback session (if session was retained in the final model). If *P*-values for the relationship between RL and startle magnitude were >0.05, no predicted values were calculated and no threshold was determined. Confidence intervals (prediction intervals) were calculated by sampling from the t-distribution and were plotted in the respective graphs on the scale of the response variable. Confidence intervals for the model coefficients (see Table S1) were calculated using the ‘confint’ function and Wald method in R.

Video data were analysed using the same procedure but applying a logistic regression model (GLM with a binomial error distribution) with startle occurrence (yes/no) as a binary response variable. Confidence intervals were calculated by using standard errors and sampling from the logistic distribution.

Rise-time data from experiment 2 were analysed using the same model selection procedure (based on AICc) and candidate variables. However, since the relationship between rise time and startle magnitude appeared to be linear for some animals, the ‘identity’ (linear) link function was tested in addition to the ‘log’ and ‘inverse’ (hyperbolic) link. In the rise-time models, focal and other animal echolocation activity was not considered as these factors did not appear to influence the threshold data.

**Fig. 4.**
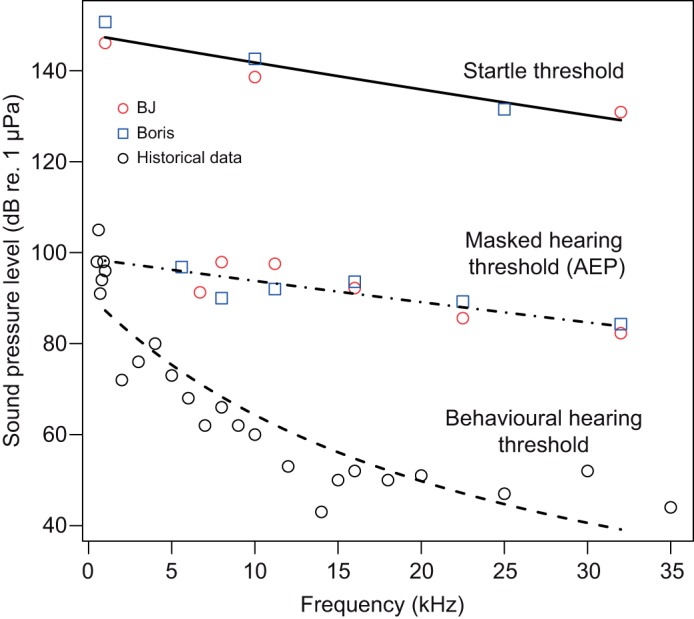
**Startle thresholds and masked AEP hearing threshold for the two bottlenose dolphins.** Trend lines represent predicted values generated by the models. A historical behavioural audiogram for a bottlenose dolphin is shown for comparison, from Johnson (1967). Trend lines represent predicted values generated by the models.

To visualize the relationship between frequency and the startle and hearing thresholds, curves were fitted to the data (Fig. 4). For this we used the same modelling approach described above, i.e. a GLM with a gamma error distribution and either a log or inverse link function using frequency as the single predictor and the thresholds as independent variables. Pseudo R-squared values (Nagelkerke) were calculated for the model that are presented in the comparison of the response metrics (Fig. 2) using the ‘modEvA’ package in R (https://cran.r-project.org/web/packages/modEvA/index.html; Barbosa et al., 2016).

## RESULTS

### Startle response characteristics

Startle flinches were clearly detected in both the video and accelerometer data. In the videos, startle responses were typically observed as a body wave or more localised flinch in the stationing dolphin (Movie 1). The accelerometer detected startle flinches reliably irrespective of the various tested tag attachment locations (Fig. 1A). The response was typically represented as an impulsive wave on all three axes of the accelerometer (Fig. 1B). The measured waveform of the startle flinch was often consistent across trials within the same playback session, particularly on the axis of the accelerometer that detected the highest response magnitudes (Fig. 1B). The p–p VeDBA within the 1 s analysis window generally exhibited a continuous increase with increasing RL (Figs 1B, 2A, 3A,B,D–H), even though there were exceptions to this general trend (Fig. 3C,I). In conjunction with this increase in startle magnitude, response latencies shortened at high RLs (Fig. 1A). The maximum norm jerk showed the same relationship, i.e. jerk increased and response latencies decreased as RLs increased (Figs 1C, 2B). Startle responses could be reliably elicited in all three test subjects but not all subjects startled at all tested RLs at all frequencies. Startle magnitude differed markedly across individuals with BJ showing the highest responsiveness, Boris intermediate and Kina low responsiveness (Fig. 3).

### Comparison of metrics used to quantify startle responses

**Fig. 5.**
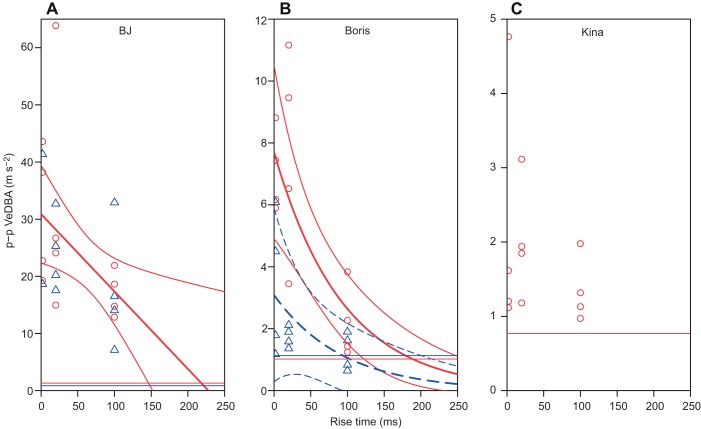
**Startle magnitude as a function of rise time in the three test subjects.** The curves represent predicted values from the model and 95% confidence intervals obtained from GLMs. The horizontal lines represent average baseline acceleration during a no-sound control for each playback session for the bottlenose dolphins BJ (A) and Boris (B), and the false killer whale Kina (C).

A direct comparison of the three metrics, p–p VeDBA, maximum norm jerk (jerk) and visual scoring of videos (‘yes/no’) is shown in Fig. 2. This comparison was conducted on the most comprehensive dataset which was obtained from BJ at the 10 kHz test frequency. The two continuous response variables showed clear correlations with RL (Fig. 2A,B). However, the confidence intervals were narrower for p-p VeDBA compared with ‘jerk’ (Fig. 2A,B). In addition, the higher pseudo *R*^2^ (Nagelkerke) for the p–p VeDBA model (87%) compared with the jerk model (77%) demonstrates that the former metric may be more suitable for quantifying startle magnitude than the latter. Both continuous response metrics performed better than the binary response variable (yes/no) obtained from scoring of the video data (pseudo *R*^2^: 73%). Hence, while some comparative aspects were retained in the following results section, p–p VeDBA was carried forward as the primary metric of choice. This is also the metric used to calculate startle thresholds at all tested frequencies (Figs 3 and 4) and to determine the effect of rise time on startle magnitude (Fig. 5).

### Factors influencing startle response likelihood and magnitude

Received level had a significant effect (*P*<0.05) on startle magnitude in 10 out of the 13 models that contained accelerometer data as a response variable. Startle response magnitude as quantified by either of the two tested metrics, i.e. the maximum p–p VeDBA or the jerk, increased with increasing RL (Figs 1B,C, 2A,B and 3). Startle magnitude did not seem to reach a ceiling level within the tested range of RLs (Figs 2 and 3). The relationship between RL and startle magnitude could be modelled reasonably well with the logarithmic or inverse link functions (Figs 2A,B and 3, Table S1). Model coefficients (β), 95% confidence intervals (CI) and *P*-values obtained from all GLMs are provided in the Table S1. These coefficients allow future studies to make predictions about the relationship between RL and startle magnitude (strength of flinch) and draw direct comparison to this paper.

The model for the jerk (Fig. 2B, BJ, 10 kHz) revealed that with each dB increase in RL, startle magnitude increased by 12.6% (e^β^=1.126, CI: 1.096/1.156, *P*<0.0001). Similarly, the model for p–p VeDBA showed that startle magnitude increased by 9.4% for each dB increase in RL (Fig. 2A, e^β^=1.094, CI: 1.076/1.113, *P*<0.0001). The response variable for the visually scored startle responses (video data, Fig. 2C) was binary and the model predicted that response likelihood increased by approximately factor 1.4 with each dB increase in RL (e^β^=1.385, CI: 1.168/1.837, *P*=0.0031).

In addition to RL, the natural logarithm of trial number or the original trial number was retained as a predictor in various models. This variable codes for habituation, i.e. a decrease in response magnitude with repeated exposures within one playback session. The natural log of trial number in the model for the p–p VeDBA model showed such a reduction of startle magnitude with each additional trial of a playback session (e^β^=0.693, CI: 0.571/0.838, *P*=0.0009). Log of trial numbers was also retained in the model for the video data and had a weak negative correlation with startle magnitude, but this narrowly missed the 0.05 significance level (e^β^=0.034, CI: 0.000/0.483, *P*=0.051). Playback session was also retained in both, the jerk model and the p–p VeDBA model but was only significant at one factor level in the latter. In the p–p VeDBA model, responses in session 3 were 31% lower than in session 1 (sessions 3: e^β^=0.693, CI: 0.492/0.976, *P*=0.0438). Coefficients pointed towards the opposite trend when comparing sessions 1 and 2 in the p–p VeDBA model but the effect had a high *P*-value (e^β^=1.124, CI: 0.772/1.633, *P*=0.5260).

The model for Boris at 10 kHz retained RL as the sole predictor and indicated an approximately 8% increase in the startle magnitude with each dB increase in RL (e^β^=1.078, CI: 1.058/1.097, *P*<0.0001). The results for Kina were unusual in that hers was the only model that retained echolocation activity in animals housed in neighbouring pens (OA) as an additional predictor, even though the relatively large confidence interval and *P*-value mean that it is not entirely clear to what extent or whether this factor really influenced startle magnitude (β=−0.304, CI: −0.623/−0.021, *P*=0.0649). The negative coefficient may hint at startle magnitude being higher when neighbouring animals were echolocating (Table S1).

The remaining models at higher and lower frequencies (1 kHz, 25 kHz, 32 kHz) showed a similar effect of RL on startle magnitude (Table S1, Fig. 3). The coefficients in these models showed that startle magnitude increased with RL (Table S1). The only models in which the *P*-values for RL were >0.05 were Boris at 32 kHz, Kina at 1 kHz and Kina at 25 and 32 kHz. Trial number or its logarithm was retained as a predictor variable in three of the remaining models (Kina 32 kHz, Boris 25 kHz, BJ 32 kHz) and was significant (*P*<0.05) in two of these models (Kina 32 kHz, Boris 25 kHz). In all cases in which trial or its logarithm was included in the model, coefficients indicated that response magnitude decreased with increasing trial number within a playback session (Table S1, Fig. 3).

### Startle thresholds

Startle thresholds that were determined by the two different accelerometer-based metrics (p–p VeDBA and jerk) yielded almost identical results (Fig. 2A,B, Table 1). BJ's threshold at 10 kHz as determined by p–p VeDBA was 139.1 dB re. 1 µPa, while her threshold as determined by maximum norm jerk was 138.1 dB re. 1 µPa. In contrast, the threshold determined by scoring of videos were significantly higher at 151.4 dB re. 1 µPa.

**Table 1.**
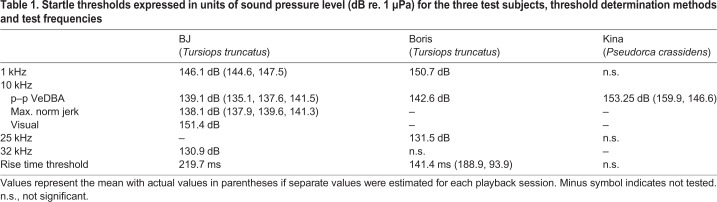
**Startle thresholds expressed in units of sound pressure level (dB re. 1 µPa) for the three test subjects, threshold determination methods and test frequencies**

The lowest threshold of 130.9 dB re. 1 µPa was measured in the ultrasonic range at a frequency of 32 kHz in BJ. Boris' threshold at 32 kHz could not be determined (RL not significant), but his response threshold at 25 kHz was 131.5 dB re. 1 µPa. Average thresholds were higher at 10 kHz, i.e. 139.1 dB re. 1 µPa in BJ, 142.6 dB re. 1 µPa in Boris and 153.3 dB re. 1 µPa in Kina. Thresholds further increased towards 1 kHz where BJ's threshold was 146.1 dB re. 1 µPa and Boris' threshold was 150.7 dB re. 1 µPa. Boris' startle threshold was 19.2 dB higher at 1 kHz than at 25 kHz while BJ's threshold was 15.2 dB higher at 1 kHz than at 32 kHz.

Startle thresholds, masked hearing thresholds obtained from AEP measurements in two of the test subjects, a historical behavioural audiogram for a bottlenose dolphin (Johnson, 1967) and trend lines (fitted/predicted values) are shown in Fig. 4. The trend lines that were fitted to the startle threshold data exhibit a slope that is intermediate between that of the masked hearing thresholds and the behavioural audiogram data. The fitted values indicate that average startle thresholds decreased by 18.2 dB from 1 kHz to 32 kHz. In contrast, the fitted values for the masked hearing thresholds only decreased by 14.1 dB in the frequency range from 1 to 32 kHz. The predicted values for the behavioural audiogram data showed a much steeper decrease by 48 dB across the same frequency range.

Our data also allow startle thresholds to be presented in units of sensation level, i.e. the level by which the sound exceeds the hearing threshold. The average sensation level across the range of tested frequencies at which a startle response occurred is 47 dB for masked AEP thresholds and 82 dB for the behavioural audiogram (Fig. 4). The modelled data in Fig. 4 indicate that the sensation level capable of eliciting a startle response at 1 kHz was 60 dB for behavioural data and 49 dB for masked AEP thresholds. The respective values at 10 kHz were 77 dB (behavioural) and 48 dB (masked AEP) while the required sensation levels at 32 kHz was 90 dB (behavioural) and 45 dB (masked AEP). Sensation levels calculated for each test subject based on linear interpolation between available audiogram values (rather than the models) showed a similar picture and are presented in Table 2.

**Table 2.**
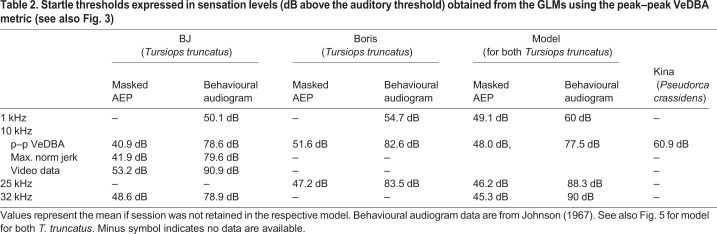
**Startle thresholds expressed in sensation levels (dB above the auditory threshold) obtained from the GLMs using the peak–peak VeDBA metric (see also Fig. 3)**

### Effects of rise time

Startle magnitude decreased with increasing rise time in all three test subjects (Fig. 5). Rise time had a significant effect on startle magnitude in BJ and Boris. For BJ, an increase in rise time by 1 ms led to a decrease in startle magnitude of 0.135 m s^−2^ (β: −0.135, CI: −0.244/−0.033, *P*=0.0164). For Kina, increases in rise time also led to a reduction of startle magnitude (see β) but the *P*-value for the variable was high (β: −0.008, CI: −0.021/0.004, *P*=0.1813). For Boris, each increase in rise time of 1 ms caused a 1.1% reduction in startle magnitude (e^β^=0.989, CI: 0.985/0.994, *P*=0.0006). The relationship between rise time and startle magnitude appeared more linear in BJ and Kina as indicated by the fact that the model with lowest AICc included the linear (‘identity’) link function. In contrast, the relationship appeared more logarithmic in Boris.

Rise time thresholds were determined as the value at which startle magnitude was expected to drop below the average ‘no sound’ control (Fig. 5). For BJ, no startle responses were expected if rise time increased beyond 220 ms, whereas for Boris, startle responses were predicted to disappear if rise times increased above an average of 141 ms (session 1: 189 ms; session 2: 94 ms).

## DISCUSSION

### Startle response characteristics

This study is the first to demonstrate the presence of the startle reflex in echolocating cetaceans and showed that the general physiological characteristics of the reflex were the same as in other mammals. Bottlenose dolphins use brief high-intensity sound pulses for echolocation and are known to possess the ability to regulate their auditory sensitivity when solving echolocation tasks (Nachtigall and Supin, 2008), e.g. they can downregulate their auditory sensitivity after a warning sound (Nachtigall and Supin, 2015; Nachtigall et al., 2016a, 2018). Since the reflex arc receives input from the cochlear nucleus, dolphins might therefore be able to suppress the startle reflex by regulating auditory sensitivity when they echolocate. The data from our study show that external sound pulses at SPLs much lower than those used in dolphin echolocation reliably trigger the startle reflex responses. This may in part be due to the absence of any predictable acoustic cues in the experimental protocol and the unpredictable playback schedule, i.e. the random delay times between the animal reaching the hoop station and the sound being presented. There was also no evidence that active echolocation behaviour influenced startle response magnitude. However, echolocation events were relatively rare and results therefore need to be interpreted with caution. Echolocation activity of animals housed in neighbouring pens was only included as a factor in one model (Kina, 10 kHz), which indicated that the acoustic environment or conspecific vocalisations did not affect startle magnitude. Kina's case is curious in that the model hinted at startle magnitude to our test stimulus being higher when neighbouring animals were echolocating, which may be related to an attention or hearing change effect. However, the factor only approached significance and no definite conclusions can be drawn.

The growth of startle magnitude with RLs and the inverse relationship between rise time and startle magnitude observed in our study are generally consistent with data on terrestrial model systems, such as rodents or humans (Blumenthal and Berg, 1986; Fleshler, 1965; Pilz and Schnitzler, 1996; Pilz et al., 1987). The significant effect of trial number and playback session in some models points towards moderate to mild within-session habituation of startle magnitude (p–p VeDBA and jerk). In addition, there is also weak evidence for habituation across sessions, as indicated by coefficients for models that retained playback session as a predictor. Rapid habituation of response magnitude over the first 10–20 trials down to a more stable baseline level is a well-known phenomenon in rats, particularly if individuals have not been extensively exposed to startling stimuli at a given frequency (Pilz and Schnitzler, 1996). Data on rats indicate that it is the slope of the input (RL)/output (startle magnitude) function that changes as the result of habituation (Pilz and Schnitzler, 1996). We did not allow for this in our models, as including an interaction between term RL and session would have been problematic given our limited data set. While latencies were not quantified in all trials, onset of the startle response typically occurred within 100–200 ms of the onset of the sound pulse (see Fig. 1B,C). This indicates that latencies were at least one order of magnitude higher than in rats (Pilz et al., 1987). This was to be expected owing to the larger size of dolphins and the longer neuronal signal transduction pathways through the spinal cord to the muscles at the tag attachment location (Fig. 1).

BJ's absolute response magnitude was much higher than Boris' which was slightly higher than Kina's (see differing scales in Fig. 3). As can be seen in Fig. 4, the differences between Boris and BJ are unlikely to be a result of differences in auditory sensitivity. However, startle response magnitude in terrestrial models is known to be influenced by genetic factors, environmental factors that influence current emotional state, and psychiatric disorders (Koch and Schnitzler, 1997; Lang et al., 1998). In addition, the fact that BJ had previously been trained for hearing control experiments while Boris was not may have played a role. Absolute response magnitude is difficult to compare because older startle chambers used in terrestrial mammals did not always provide calibrated accelerometer data, and more importantly, tag attachment location and body size influence acceleration signals in tagged marine mammals. However, the highest jerks observed in our study (e.g. 2401 m s^−2^ in BJ at 32 kHz; RL:162 dB re. 1 µPa) were comparable in magnitude to rapid jaw movements observed in prey capture attempts in harbour seals (*Phoca vitulina*) (Ydesen et al., 2014).

### Comparison of response metrics

In our study, p–p VeDBA performed better as a response variable for startle magnitude than the maximum norm jerk (as defined by Ydesen et al., 2014). This can be seen in the narrower confidence intervals and higher pseudo *R*^2^ in the VeDBA model for BJ at 10 kHz (compare Fig. 2A and B). We have therefore presented most of the derived analysis (e.g. thresholds and effect of rise time) using the p–p VeDBA metric. However, we believe that this result should not be over-interpreted, and the jerk metric (which performs relatively well) may have a role to play in future studies. Our study design involved animals stationing in a hoop with relatively little fluking motion. This meant that distinct peaks in the acceleration record could be easily detected above noise. However, the situation will be different in studies that investigate the effects of anthropogenic stressors (e.g. impulsive noise) on wild tagged animals. It is possible that the jerk will perform better in these conditions as it is based on the norm of the differential of acceleration and may therefore be more resistant to steady state movement noise.

### Startle thresholds and sensation levels

The fact that the startle threshold roughly followed the hearing threshold of the dolphins across a range of different frequencies is also consistent with data on terrestrial model systems (Pilz et al., 1987). A comparison of absolute thresholds to terrestrial model systems is complicated by the fact that caution must be exercised when comparing SPLs in air and water. This is only partly due to the different reference sound pressure conventions (1 µPa in water versus 20 µPa in air) but also the result of the differing impedances of the two media (Au, 1993). Hence, we used sensation levels, i.e. the level by which a stimulus exceeds the auditory (detection) threshold/hearing threshold (audiogram) of the test subject for comparisons.

The average startle threshold in our study was only 47 dB above the masked auditory AEP threshold (Fig. 4). Human eyeblink responses, which are mediated by a similar neuronal pathway, have been shown to occur at sensation levels as low as 50–70 dB (Blumenthal and Berg, 1986). However, sensation levels based on AEP thresholds in our study were considerably lower than data for any other mammal (Pilz et al., 1987). There are three possible explanations for this discrepancy: (1) the hearing thresholds were masked due to the noise caused by snapping shrimp in the test pens (Au and Banks, 1998) and therefore actual hearing thresholds under quiet conditions would be lower and sensation levels higher; (2) hearing thresholds were obtained with an electro-physiological method which typically yields thresholds that are approximately 20 dB higher than those obtained with traditional psycho-physical methods (see Szymanski et al., 1999 for a comparison of AEP and behavioural methods); and (3) the test subjects in our study showed some age-related hearing loss at higher frequencies. We suggest that the main factor here was masking noise during AEP measurements since startle thresholds exceeded the historical behavioural audiogram (Johnson, 1967) within the tested range of frequencies on average by 82 dB (fitted values, Fig. 5). This is remarkably close to data from terrestrial model systems, where clear startle responses occurred when an acoustic stimulus exceeded the auditory threshold by 75 dB (Ouagazzal et al., 2006) to 87 dB (Pilz et al., 1987).

The sensation level data based on the behavioural audiogram (Johnson, 1967) suggest that stimuli with somewhat lower sensation levels can startle at low frequencies compared with higher frequencies (Fig. 4, Table 2). This effect is akin to the flattening of equal loudness contours towards the edge of the hearing range at high SPLs, which is known from studies on humans (Fletcher and Munson, 1933) and harbour porpoises (Wensveen et al., 2014). However, this trend is not matched by masked AEP threshold data for the two bottlenose dolphins where the audiogram and startle threshold lines ran more or less parallel and the sensation level capable of eliciting startle were similar across the whole frequency range (Fig. 4). This is in line with studies on rats where startle and hearing thresholds also ran parallel (Pilz et al., 1987). However, given the small data set and low numbers of individuals tested, this result should not be over-interpreted.

Startle thresholds have been measured in few marine mammals and all previous data are based on visual scoring of responses. A study on phocid seals found an average pure tone threshold of 159 dB re. 1 µPa (at 1 kHz) and a sensation level of ∼93 dB (Götz and Janik, 2011). In the present study, visual scoring of videos resulted in a startle threshold for BJ that was 12.3 dB higher compared with the standard accelerometer-based metric. This is probably because the weak muscle flinches were not visible on a video but could still be detected above baseline movement activity using acclerometry. Hence, actual pure tone startle thresholds for phocid seals may be closer to 147 dB re. 1 µPa. Kastelein et al. (2012) used movement responses in free-swimming harbour porpoise as a proxy for what they thought were startle responses and found responses at very low SPLs (down to 99 dB re. 1 µPa at high frequencies). However, their sounds had long rise times of 50 ms and the authors included responses that occurred after the 1 s long stimulus ceased. Hence, it is likely that responses reported by Kastelein et al. (2012) included non-startle responses such as orienting or defence responses (Turpin et al., 1999). Long rise times are less likely to cause startle responses, so the terminology in Kastelein et al. (2012) is most likely not correct.

Another factor that needs to be considered when comparing thresholds and startle magnitude is sound type and stimulus bandwidth. Broadband noise is a more potent startle stimulus than a pure tone as noise triggers responses at much lower amplitudes and causes a higher startle magnitude than a pure tone presented at the same stimulus amplitude (Stoddart et al., 2008). Our stimuli in experiment 1 (1/3 octave band noise pulses) were probably more potent than pure tones used in earlier studies that found thresholds at higher sensation level (Götz and Janik, 2011; Pilz et al., 1987). Broadband stimuli like those we used in the rise time experiment or that were used in rodent studies (Stoddart et al., 2008) are likely to be even more effective.

### Rise times

Startle magnitude decreased monotonously in all three test subjects with increasing rise time. A study investigating eyeblink responses in humans using broadband noise stimuli also showed that startle magnitude decreased linearly with increasing stimulus rise time (Ramirez et al., 2005). The fact that the model for Boris included a logarithmic link function may point towards a more variable relationship but should not be overinterpreted as only three rise-time values were tested.

Stimuli with the longest rise time tested in our study (100 ms) still occasionally induced faint startle responses in Boris and BJ. Our models suggest that such responses should disappear completely if rise time increased beyond 220 ms for BJ and 141 ms for Boris. Early studies on rats found that a stimulus had to reach its maximum amplitude within 12 ms of its onset to elicit a startle response (e.g. Fleshler, 1965). This stringent view on the necessity of short onset times needs to be revised in light of both our data on odontocetes and later studies on rats, which showed that pure tones presented at 80–90 dB re. 20 µPa were capable of eliciting weak startle responses even if rise times were 100 ms long (Ison, 1978). Broadband noise initiates higher startle magnitudes at the same RLs in comparison to pure tones (Stoddart et al., 2008). Hence, if sensation levels are high, broadband stimuli can still elicit weak responses at rise times of 100 ms (Ramirez et al., 2005).

### Implications for mitigating the effects of noise on marine mammals

The startle reflex has previously been shown to induce flight responses, interrupt foraging behaviour and cause sensitisation of subsequent avoidance behaviour in the majority of tested grey seals (Götz and Janik, 2011). This effect has been used successfully to keep seals from approaching and predating on fish farms (Götz and Janik, 2015, 2016). Bottlenose dolphins are reported to refuse to pass a sound source when stimuli with a 50 ms rise time exceeded sensation levels beyond the startle threshold (Houser et al., 2013). Houser assumed that stimuli with a rise time as long as 50 ms could not elicit the startle reflex in dolphins. However, our results demonstrate that the relationship between rise time and startle magnitude is more gradual in dolphins, which has also been found in an earlier study on rats (Ison, 1978). While the dolphins in our study did not show avoidance, one dolphin also initially backed out of the hoop station during exposure and this behaviour occurred multiple times in a pilot study conducted before the actual experiment. In contrast to animals used by Houser et al. (2013), our animals had been part of noise exposure trials before and were trained to remain on station when presented with sound. This may explain why they did not break station when hearing startle sounds. Further studies are needed to investigate whether untrained dolphins show flight and avoidance responses when exposed to startling stimuli or even sensitize, as has been found in some seals (Götz and Janik, 2011). If this is the case, then marine mammal noise exposure criteria for behavioural responses (e.g. Southall et al., 2007), which are commonly used by regulators to inform policy decisions should consider rise time as an additional predictor.

Deep-diving beaked whales have been shown to strand as a result of exposure to mid-frequency military sonar signals with pathology indicating gas bubble lesions (Jepson et al., 2003). It has been suggested that these strandings occur as the result of an extremely long-distance movement or flight response, mild forms of which have also been observed in controlled exposure experiments (Tyack et al., 2011). This particular sensitivity of beaked whales to mid-frequency sonar may be surprising given that sonar signals do not fall in their most sensitive range of hearing (Pacini et al., 2011). Our results indicate startle responses can still be elicited in odontocetes at moderate RLs at mid frequencies (e.g. BJ's threshold at 10 kHz was only ∼139 dB re. 1 µPa). Furthermore, response magnitude increases logarithmically or hyperbolically with RL. Hence, it is possible that extreme startle responses as a result of temporary exposure to high RLs play a role in such strandings (Harris et al., 2018). If the startle reflex is one of the underlying mechanisms, noise effects could be mitigated by increasing the rise time of navy sonar signals.

### Conclusions

Our study demonstrated that the startle reflex is conserved in two species of echolocating and fully aquatic mammals. The basic physiology followed similar principles as in terrestrial mammals, i.e. startle magnitude was positively correlated with SPL and negatively correlated with rise time. Average startle thresholds expressed as sensation levels (i.e. the level above the hearing threshold) were surprisingly similar to those of terrestrial mammals (∼82 dB), in spite of the physical differences of their environments. Hence, startle thresholds seem to have evolved in relation to auditory detection thresholds. Our results further support the hypothesis that the startle reflex evolved early in the mammalian lineage and remained largely unchanged in a wide variety of taxa. It is interesting that selection pressures driving anatomical and neurophysiological adaptations to aquatic hearing and echolocation in odontocetes do not seem to have led to significant changes in the basic functioning of this reflex arc. The functioning and physiological principles of the startle reflex should be taken into account when developing guidelines and mitigation methods regarding the effects of anthropogenic noise on echolocating marine mammals.

## Supplementary Material

Supplementary information
