# Supplementary material for: The startle reflex in echolocating odontocetes: basic physiology and practical implications
Source: J Exp Biol. 2020 Mar 12;223(5):jeb208470. doi: 10.1242/jeb.208470 (PMC7075047; doi:10.1242/jeb.208470)
Supplement: Supplementary information [file jexbio-223-208470-s1.pdf]

Supplementary material

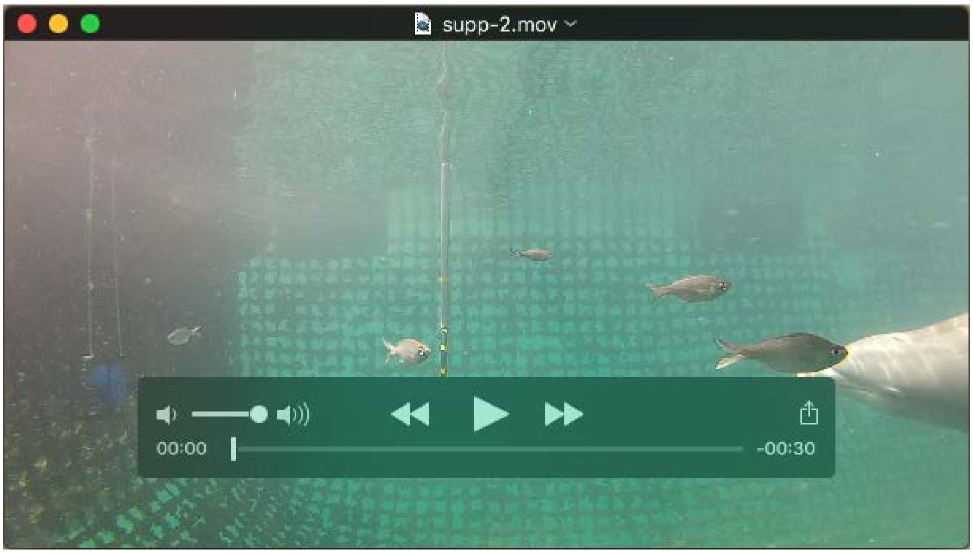

Movie 1: Animal in hoop station exhibiting a startle response

Table S1: Full record of model specifications (retained predictor variables/factors and link function), model coefficients, associated 95% confidence intervals (CIs) and p-values. The interpretation of the coefficients depends on the link function of the model. In a GLM with a linear link function (identity) $Y = \beta_0 + \beta_1 x_1 + \ldots \beta_n x_n$ where the response Y is modelled by n explanatory variables the coefficient β_n are to be interpreted additively, i.e. with each one increment in the predictor variable x_n the response Y will increase by β_n. In a model with a logarithmic ($\log_e$) link function $Y = \beta_0 + \beta_1 * \ln(x1) + \ldots \beta_{n*} \ln(x_n)$ the exponentiated coefficients $e^{\beta n}$ are to be interpreted multiplicatively, i.e. with each one increment increase in the predictor variable x_n the response Y will increase by factor $e^{\beta n}$ (i.e. the response will be e^{β} times higher). In a logistic regression model e^{β} represents a multiplicative increase in the response likelihood.

**BJ 10kHz: VedBa (p-p VeDBA)**				
**Link: Log_e**	Coefficient e^{β}	CI 5%	CI 95%	P values
**(Intercept)**	0.000	0.000	0.000	<0.0001
**RL**	1.094	1.076	1.113	<0.0001
**Session 2**	1.124	0.772	1.633	0.5260
**Session 3**	0.693	0.492	0.976	0.0438
**log(trial number)**	0.693	0.571	0.838	0.0009
**BJ 10kHz: Maximum norm jerk**				
**Link: Log_e**	Coefficient e^{β}	CI 5%	CI 95%	P values
**(Intercept)**	0.000	0.000	0.000	<0.0001
**RL**	1.126	1.096	1.156	<0.0001
**Session 2**	1.577	0.868	2.860	0.1419
**Session 3**	0.733	0.414	1.301	0.2946
**BJ 10kHz: Video data (GLM with binomial error distribution, logistic regression model)**				
**Link: Logistic**	Coefficient e^{β}	CI 5%	CI 95%	P values
**(Intercept)**	0.000	0.000	0.000	0.0039
**RL**	1.385	1.168	1.837	0.0031
**log(trial number)**	0.034	0.000	0.483	0.0509
**BJ 1kHz: p-p VeDBA**				
**Link: Identity**	Coefficient β	CI 5%	CI 95%	P value
**(Intercept: session 1)**	-93.646	-131.772	-61.193	<0.001
**RL: session 1**	0.658	0.444	0.914	<0.001

Journal of Experimental Biology • Supplementary information

Link: Inverse	Coefficient β	CI 5%	CI 95%	P value
(Intercept: session 2	6.227	2.897	10.657	0.0091
RL: session 2	-0.038	-0.065	-0.017	0.0110
**BJ 32kHz: p-p VedBA**				
Link: Inverse	Coefficient β	CI 5%	CI 95%	P values
(Intercept)	2.688	6.680	34.153	<0.0001
RL	-0.017	0.979	0.988	<0.0001
Log_e (trial number)	0.013	1.001	1.028	0.079
**Boris 10kHz: p-p VedBA**				
Link: Log_e	Coefficient e^{β}	CI 5%	CI 95%	P values
(Intercept)	0.000	0.000	0.000	<0.0001
RL	1.078	1.058	1.097	<0.0001
**Boris 25kHz: p-p VedBA**				
Link: Log_e	Coefficient e^{β}	CI 5%	CI 95%	P values
(Intercept)	0.006	0.000	0.109	0.0069
RL	1.035	1.014	1.056	0.0074
Log_e (Trial number)	1.448	1.136	1.829	0.0110
**Boris 32kHz: p-p VedBA**				
Link: Log_e	Coefficient e^{β}	CI 5%	CI 95%	P values
(Intercept)	0.491	0.001	588.828	0.8331
RL	1.011	0.964	1.059	0.637
**Boris 1 kHz: p-p VedBa**				
Link: Inverse	Coefficient β	CI 5%	CI 95%	P values
(Intercept)	3.983	2.316	5.718	0.0001
RL	-0.022	-0.033	-0.012	0.0004
**Kina 10 kHz: VedBA**				
Link: Inverse	Coefficient β	CI 5%	CI 95%	P value
(Intercept)	4.489	2.827	6.305	0.0001

Journal of Experimental Biology • Supplementary information

**RL**	-0.023	-0.035	-0.013	0.0010
**OA**	-0.304	-0.623	-0.021	0.0649
**Kina 25kHz: p-p VedBA**				
**Link: Log_e**	Coefficient e^{β}	CI 5%	CI 95%	P values
**(Intercept)**	12.618	0.562	293.843	0.1289
**RL**	0.985	0.964	1.006	0.170
**Kina 32kHz: p-p VedBA**				
**Link: Log_e**	Coefficient e^{β}	CI 5%	CI 95%	P values
**(Intercept)**	0.169	0.013	2.214	0.2293
**RL**	1.020	1.002	1.038	0.0731
**Log_e (Trial number)**	0.932	0.882	0.983	0.0294
**Kina 1kHz: p-p VedBa**				
**Link: Inverse**	Coefficient β	CI 5%	CI 95%	P value
**(Intercept)**	-0.154	0.128	6.251	0.8792
**RL**	0.006	0.993	1.018	0.3809
**Rise time model: Boris**				
**Link: Log_e**	Coefficient e^{β}	CI 5%	CI 95%	P value
**(Intercept)**	7.707	5.607	10.886	<0.0001
**RT**	0.989	0.985	0.994	0.0002
**sess2**	0.401	0.268	0.600	0.0002
**Rise time model: BJ**				
**Link: Identity**	Coefficient β	CI 5%	CI 95%	P value
**(Intercept)**	30.886	23.987	40.369	0.0000
**RT**	-0.135	-0.244	-0.033	0.0164
**Rise time model: Kina**				
**Link: Identity**	Coefficient e^{β}	CI 5%	CI 95%	P value
**(Intercept)**	2.190	1.505	3.299	0.0005
**RT**	-0.008	-0.021	0.004	0.1813

Threshold plotting models: startle thresholds				
**Link: Log$_e$**	Coefficient e^{β}	CI 5%	CI 95%	P value
**(Intercept)**	147.957	144.602	151.411	<0.0001
**Frequency (kHz)**	0.996	0.994	0.997	0.0031
**Threshold plotting models: Masked AEP Threshold**				
**Link: Log$_e$**	Coefficient e^{β}	CI 5%	CI 95%	P value
**(Intercept)**	98.725	95.136	102.468	<0.0001
**Frequency (kHz)**	0.995	0.993	0.997	0.0006
**Threshold plotting models: Behavioural audiogram (Johnson, 1967)**				
**Link: Inverse**	Coefficient β	CI 5%	CI 95%	P value
**(Intercept)**	0.011	0.01	0.012	<0.0001
**Frequency (kHz)**	0.0005	0.0004	0.001	<0.001

Journal of Experimental Biology • Supplementary information
